# Chemokine Ligand-3 (CCL3) as a Novel Mediator of Inflammatory Bowel Disease Activity

**DOI:** 10.1016/j.gastha.2026.101081

**Published:** 2026-08-04

**Authors:** Eileen O’Brien, Kyra Fine, Olimpia Sienkiewicz, Yuhong Wei, Christine E. Orr, Daniel J. Mulder

**Affiliations:** 1 Department of Biomedical and Molecular Sciences, Queen’s University, Kingston, Ontario, Canada; 2 Gastrointestinal Diseases Research Unit, Queen’s University, Kingston, Ontario, Canada; 3 Goodman Cancer Institute, McGill University, Montreal, Quebec, Canada; 4 Department of Pathology and Molecular Medicine, Queen’s University, Kingston, Ontario, Canada; 5 Department of Pediatrics, Queen’s University, Kingston, Ontario, Canada

**Keywords:** Inflammatory Bowel Diseases, Inflammation, Chemotaxis, Chemokines, Mass Cytometry, CCL3

## Abstract

**Background and Aims:**

Inflammatory bowel disease (IBD) is characterized by dysregulation of the immune system, driven in part by pro-inflammatory macrophage polarization. Chemokine ligand-3 (CCL3) is a chemokine known to mediate inflammation and act to polarize M1 macrophages at the site of inflammation. CCL3 is upregulated in patients with IBD, but its contribution to disease pathophysiology beyond that is unknown. Our hypothesis was that CCL3 expression increases at sites of more intense inflammation in biopsies from patients with IBD and it mediates inflammation through neutrophils and M1 macrophages.

**Methods:**

Gastrointestinal mucosal biopsies from patients with IBD in varying states of inflammation were immunostained for CCL3 and co-immunostained for C-C motif chemokine receptor 1 (CCR1) and C-C motif chemokine receptor 5 (CCR5). Imaging mass cytometry was performed to identify CCL3-expressing cell spatial relationships.

**Results:**

Immunohistochemistry found that CCL3 was expressed in a variety of cell types in patients with both ulcerative colitis and Crohn’s disease. CCL3 receptors, CCR1 and CCR5, were widely expressed as well, with co-expression of CCL3 occurring in a subset of these cells that was higher than that of CCL3/CCR5. Imaging mass cytometry highlighted a positive correlation between CCL3 and the severity of inflammation of IBD patient biopsies. The majority of CCL3-positive cells were neutrophils and macrophages (in particular, proinflammatory M1 macrophages), but there was also expression on eosinophils and epithelial cells.

**Conclusion:**

CCL3 is expressed in many cell types in the gut mucosa in patients with IBD and is positively associated with the severity of local inflammation. CCL3-mediated effects in IBD may occur primarily through the CCR1 signaling pathway, leading to attraction and polarization of macrophages and neutrophils.

## Graphical Abstract

**Figure:**
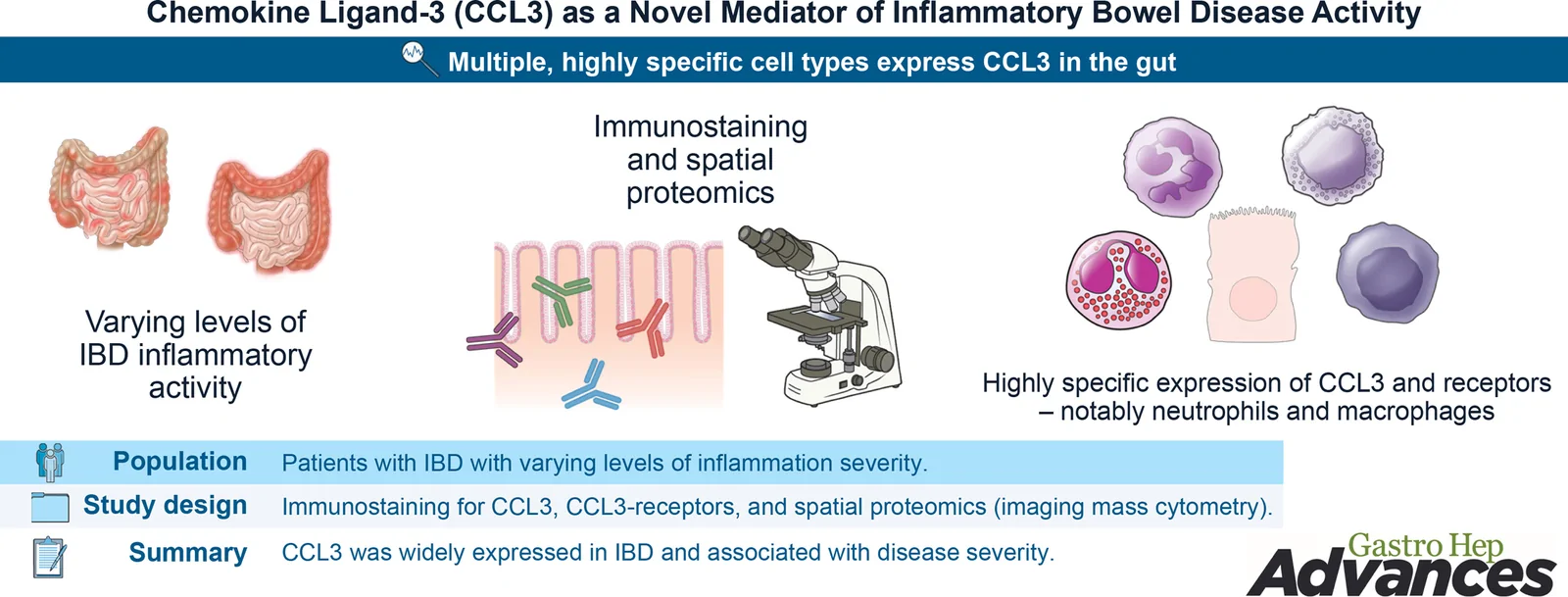

## Background

Inflammatory bowel disease (IBD) is a chronic lifelong autoinflammatory condition affecting the gastrointestinal (GI) tract. It is subdivided into ulcerative colitis (UC) and Crohn’s disease (CD). Immunologically, IBD is characterized by a dysregulation in the mucosal immune system leading to a chronic inflammatory response and damage to the GI epithelial barrier.^1^ Patients can experience acute unexplained exacerbations of inflammation characterized by inflammatory cell infiltrate, including neutrophil and macrophage infiltration into the gut mucosa, despite targeted therapy.^2^ New biomarkers that reflect neutrophil and macrophage activity could allow for earlier diagnosis of exacerbations and improve targeted therapy. There are currently no approved therapies that specifically target neutrophils or macrophages, despite their early role in the acute inflammatory response.^3^

Chemokine ligand-3 (CCL3) has been identified to have a potential role in mediating IBD exacerbations.^3^ CCL3 is a cytokine produced by a variety of immunologically active cell types, mostly neutrophil and monocyte-lineage cells.^4^ CCL3 acts by binding to its specific receptors, C-C motif chemokine receptor 1 (CCR1) and C-C motif chemokine receptor 5 (CCR5), and subsequently induces chemoattraction of many inflammatory cell types, namely macrophages, monocytes, neutrophils, natural killer cells, dendritic cells, and T lymphocytes.^4^ These broad effects on both innate and adaptive immune activity could mean it is an early mediator of inflammation in IBD.

Previous studies have found that CCL3 expression is higher in the colons taken from mice in an experimental colitis model^5^ and in UC patients^6^ and that CCL3 gene expression is increased in cases of active UC.^7^ Furthermore, CCL3 gene expression is higher in M1 than M2 macrophages in UC patients and in specific neutrophil populations.^8^ However, there remain gaps in understanding the role of CCL3 in IBD, in particular, if the gene expression changes previously described translate to protein expression changes and if the receptors of this chemokine are present in the gut.

The goal of this study was to characterize CCL3 protein expression in the gut mucosa in the range of inflammatory states in IBD. We hypothesized that CCL3 expression increases at sites of more intense inflammation in biopsies from patients with IBD and it mediates inflammation through neutrophils and M1 macrophages.

## Methods

### Study Population

GI mucosal biopsies and clinical data were obtained from patients in our regional IBD database at the Kingston Health Sciences Centre in Ontario, Canada (HSREB 6033229). All authors had access to the study data and have reviewed and approved the final manuscript. Patients in clinically active and remissive states of IBD were included. Active and remission states were determined by the clinical pathology report and further quantified by the Naini and Cortina score.^9^ After scoring, activity scores were confirmed to correspond accordingly to the activity level in the pathologist’s report. For each set of experiments, a different cohort of IBD patients was included due to the limited biopsy tissue available for each experiment. Regions of representative inflammation in the lamina propria were selected for imaging, generally representing areas where the inflammation was most intense while the underlying tissue ultrastructure was clearly discernable. Regions of inflammation included cryptitis, crypt abscesses, and cell-dense areas in the lamina propria.

For immunohistochemistry (IHC) experiments, all patient gut mucosal biopsies used were from adult participants (mean age 38 years old). There were a total of 24 biopsies from 9 patients with UC and 21 coming from 7 separate patients with CD. Of this cohort, 15 biopsies had no features of acute or chronic inflammation. All biopsies’ severity of inflammation was scored using the Naini and Cortina score. Cohort demographic details, medication at time of their endoscopy, Naini and Cortina score, and IBD subtype are summarized in Supplementary Table 1A.

For coexpression immunofluorescence (IF) experiments, 11 gut mucosal biopsies of 10 IBD pediatric patients (mean age, 13 years old) were selected. This cohort had 5 patients with no pathology features of acute or chronic inflammation and 6 with features of acute and/or chronic inflammation. There were 7 patients with CD and 4 with UC. Cohort demographic details, medication at the time of their endoscopy, Naini and Cortina score, and IBD subtype are summarized in Supplementary Table 1B.

For imaging mass cytometry (IMC) experiments, 2 1 mm by 1 mm regions of interest (ROIs) were selected from gut mucosa biopsies of 10 IBD patients. ROIs were selected that (1) were representative of the overall biopsy, (2) if possible, were oriented along the vertical axis of crypts, and (3) included both lamina propria and epithelium. If there was variability in the degree of inflammation within a biopsy, the most inflamed region was selected. As displayed in Supplementary Table 1C, there were 4 adult patients (mean age = 36) and 26 pediatric patients (mean age = 14) in this cohort. This cohort included 8 patients with UC and 22 with CD. Fifteen biopsies had pathologic features of acute and/or chronic inflammation, whereas the other 15 had no signs of acute or chronic inflammation.

### Tissue Processing and Inflammation Scoring

GI mucosal biopsies for all experiments were formalin fixed embedded in paraffin (FFPE) for preservation and were sectioned at 4 μm thickness. Slides for inflammation scoring were stained with hematoxylin and eosin. Slides were scored based on degree of acute and chronic inflammation using the Naini and Cortina score, which aims to quantify the probability of a diagnosis of IBD.^9^ It is also allowed for semi-quantitative comparison of ileum and colon scores. This provided an advantage over other scoring methods when considering the importance of both the ileum and colon in IBD. This score evaluates architectural and crypt distortion, cryptitis, crypt abscesses, erosions/ulcers, and granulomas based on a cumulative scale. The score values limiting inter-user variability, as it focuses on the cumulation of the highly variable features of inflammation to form a score. Biopsy identities were blinded for all scoring and experimentation. Scoring was compared with pathologists’ descriptive assessments of the inflammation in each biopsy, summarized in Supplementary Figure 1.

### Pathologic Features of Inflammation

Pathology reports for each biopsy were reviewed and scored in 3 categories: active inflammation, chronic inflammation, and presence of any inflammation (either active or chronic inflammation or both). Disease activity was classified as “none,” “mild” (including cryptitis), “moderate” (including crypt abscess), and “severe” (including ulceration). Chronic inflammation—crypt architectural distortion, Paneth cell metaplasia, and lymphoplasmacytic inflammation—was classified as “present” or “absent.” Presence of any inflammation—either acute or chronic inflammation—was classified as “present” or “absent.” These 3 categorical features were used for statistical analysis throughout.

### Immunohistochemistry

Gut mucosal biopsies were deparaffinized using a xylene/ethanol gradient. Heat-induced antibody retrieval was done by placing slides in commercial Antigen Retrieval Solution (Abcam) heated to 100 °C and then kept in a high-humidity chamber for 20 minutes, then cooled for 25 minutes on ice. Biopsies were treated with a horseradish peroxidase (HRP) inhibitor, a protein inhibitor, then an HRP conjugate, and a 3,3′-diaminobenzidine (DAB) substrate with Rabbit Specific HRP/DAB Detection IHC kit (Abcam) as per the manufacturer’s instructions. Antibody concentration was optimized using serial dilution and positive lung and tonsil tissue controls. The primary antibody stain with rabbit antihuman CCL3 was 1/500 dilution. Further concentrations and dilutions are available in Supplementary Table 2. Absence of overstaining and nonspecific staining was monitored with confirmed negative staining of smooth muscle cells, negative staining in negative controls without a primary antibody added, and expected individual cell type staining patterns based on images from previous studies.^10^^,^^11^ Image acquisition was done with an Olympus BX60 microscope.

Images of IHC-processed biopsies were imported into QuPath (version 0.4.4) for further analysis. The area of the lamina propria of biopsies was manually bounded for further cell quantification of this region alone. CCL3-positive cells were detected using the “Positive Cell Detection” tool in QuPath. Parameters for positive cell detection can be found in Supplementary Table 3. The threshold for positive DAB staining was set as the value that split the large and shallow peaks of the histogram that corresponded with the stain color.

### Immunofluorescence

In the same way as IHC, biopsies were deparaffinized with a xylene/ethanol gradient, and heat-induced antibody retrieval was performed. The tissue was treated with 5% donkey serum (Abcam) for 60 minutes to block nonspecific binding. Biopsies were then incubated overnight with primary antibodies: rabbit-raised antihuman CCL3 at 1/500 dilution, goat-raised antihuman CCR1 at 1/500 dilution, and mouse-raised antihuman CCR5 at 1/60 dilution. Slides were washed and incubated with secondary fluorescent-tagged antibodies for donkey anti-rabbit IgG Alexa Fluor 647 at 1/400 dilution, donkey anti-goat IgG Alexa Fluor 488 at 1/400 dilution, and donkey anti-mouse IgG Alexa Fluor 555 at 1/200 dilution. Further details of the antibodies used are available in Supplementary Table 2. Finally, mounting medium containing 4′,6-diamidino-2-phenylindole was added.

Light and immunofluorescent images for both IHC and IF were captured on a Leica Mica Microhub confocal and widefield fluorescent microscope. For IF experiments, 5 ROIs per gut biopsy were captured at 20x magnification. Microscope parameters were optimized and set for all images. Intensity was measured through ImageJ (version 1.54). The value of the mean intensity +2 standard deviations for each channel of the negative control slides was subtracted from experimental biopsy ROIs. Gaussian smooth filter α = 1 was applied to eliminate background noise. Triangle thresholding was applied to all ROIs. Triangle thresholding was selected over other thresholding methods because of the unimodal nature of the ROI intensity histograms (the Otsu method is better suited for bimodal, whereas Triangle is better suited for unimodal). The fluorescent intensity for each biopsy was calculated as the mean intensity of the 5 representative ROIs per biopsy.

### Colocalization Measurement

The same 5 ROIs were analyzed to determine the colocalization of CCL3 with its receptors CCR1 and CCR5. The ROIs were captured on the Leica Mica Microhub confocal and widefield fluorescent microscope at 63x magnification. Microscope parameters were optimized and then set the same for all images. To control for nonspecific staining, the value of the mean intensity +2 standard deviations for each channel of the negative control slides was subtracted from experimental biopsy ROIs. ImageJ’s Coloc2 (version 3.10) was used to measure Mander’s coefficient of co-occurrence and Spearman’s ranked coefficient of correlation. Mander’s M1 coefficient measures the portion of CCL3 fluorophores that overlap with CCR1 or CCR5 fluorophores.^12^ The M1 coefficient is of particular interest to the study, as we wanted to know the proportion of CCL3-positive pixels that coincided with the receptors. Costes’ thresholding method was used for threshold intensity measurements for co-occurrence measurement. Colocalization values for each biopsy were calculated as the mean co-occurrence and correlation of 5 ROIs per biopsy.

### Sample Staining and Imaging Mass Cytometry Acquisition

IMC was used to profile the co-expression of multiple proteins simultaneously and map their spatial relationships at single-cell resolution. An optimized 33 metal-conjugated antibody cocktail was applied to formalin-fixed paraffin-embedded GI tissue biopsy slides. The details of the IMC antibodies used are presented in Supplementary Table 4. Antibody optimization, sample staining, and IMC acquisition were conducted by the Single Cell and Imaging Mass Cytometry Platform at McGill University, according to their published protocol.^13^ Imaging acquisition was performed using the Standard Biotools (formerly Fluidigm) Hyperion Imaging System at a resolution of 1 μm/pixel and a laser frequency of 200 Hz, in accordance with the manufacturer’s specifications.

### Imaging Mass Cytometry Data Processing and Cell Segmentation

Image preprocessing and analysis steps were adapted from the Bodenmiller group’s Multiplex Imaging Pipeline.^14^ This protocol utilizes Python (version 3.11) packages *readIMC* (v0.4) and *ImcSegmentationPipeline* (v1.0) to process the raw IMC data files, compiling spatial relationships, and quantifying protein expression data for each channel into labeled relational data within .csv files.

Deep learning cell segmentation was performed using Ilastik (v1.4.0) to convert each cell into its own data object for further analysis. Markers for DNA1, DNA2, CD3, CD20, CD68, CD15, E-cadherin, and pan-cytokeratine were used to segment cells. This approach was used because Hunter et al^15^ have shown that nuclei-only detection underrepresented cell types and recommended including cytoplasm cell type-specific markers. CD3, CD20, CD68, CD15, E-cadherin, and pan-cytokeratine denote the 5 main cell types of interest: T-cells, B-cells, macrophages, neutrophils, and epithelial cells. After manually identifying a subset of cell nuclei and cytoplasm, Ilastik was used to interpret the gray pixel intensity and built a random-forest machine learning model to classify individual cells. Quality of cell segmentation was manually monitored throughout the process by the reconstructed image analysis and probability maps generated by the program and then manually refined by pixel-based classification.

Cell Profiler (v4.2.8) then used the generated probability maps of segmented cells to create cell “masks.” These masks represented the computational borders of a cell from which the expression data of each channel are quantified. Next, a data frame was compiled that included the expression data for each marker within a cell, the size parameters of the cell, and the cell coordinates. This data frame was then used for further downstream spatial analysis. Cell segmentation quality was reviewed by evaluating reconstructed images, such as those seen in Figure 14, using the R packages *SpatialExperiment* (v1.14.0) and *cytomapper* (v1.16.0).

Using the Bodenmiller group’s multiplex image downstream processing workflow, a series of quality control steps were followed.^14^ First, the quality of the cell segmentation was assessed by visual analysis of the generated segmented tissue image with the labeled cell boundaries, using the R *Cytomapper* package (v1.16.0). The steps for quality control included: arcsinh transformation to distinguish between positive and negative expression, Otsu’s thresholding method to control for background staining, and manual elimination of cells smaller than 5 px^2^.

For cell subtype clustering, an unsupervised gating approach was used because it introduces the least amount of human interference, effectively minimizing bias.^16^ We performed k-means nearest neighborhood clustering due to its advantage in modeling functional for multidimensional data like that present in this study. To identify the parameters needed for k-means clustering, we calculated the neighborhood purity and silhouette width metrics from the R *bluster* package (v1.14.0). We then compared multiple parameter permutations as seen in Supplementary Figure 3. The neighborhood purity metric evaluates the proportion of observations in a cluster compared to the proportion of those observations in another cluster. A high purity means that there are few observations in other clusters that are similar to the observations in the cluster being evaluated. The silhouette width metric evaluates how well clusters are separated based on the distance between clusters and on how close individual nodes are within a cluster. A high silhouette width indicates that clusters are effectively separated. Neighborhood purity and silhouette width identified k = 20 and type = rank as the most optimal clustering parameters. Cell phenotypes were assigned to each cluster based on cell marker expression, Supplementary Table 5.

### Statistical Analysis

Statistical analysis and graph formatting were performed in R using the packages *dyplr* (v1.1.4) and *ggplot2* (v3.5.2). Histogram evaluation and the Shapiro-Wilks test were used to determine if the data were normally distributed, as seen in Supplementary Figure 4. Nonparametric Wilcoxon-signed rank tests were used to compare the percent of CCL3-positive cells between subtypes and disease activity states; mean fluorescent intensities between subtypes and disease activity states; mean fluorophore co-occurrence and correlation between subtypes, activity states, and receptors; and mean CCL3 expression between subtypes and disease activity states. Nonparametric Kruskal-Wallis tests paired with Dunn’s multiple comparisons tests were used to compare mean CCL3 expression between inflammatory activity states. Spearman’s ranked coefficient was used to evaluate the correlation between the Naini and Cortina score, the percent of CCL3-positive cells, mean CCL3 expression/intensity, CCR1 intensity, and CCR5 intensity. Heatmaps of cell marker expression levels were built using R package *dittoSeq* (v1.16.0). Cell expression graphs were created using the R packages *imcRtools* (v1.10.0). UMAP was evaluated using the R package *scran* (v1.32.0). Spatial visualization of IMC tissues was performed using the R packages *SpatialExperiment* (v1.14.0) and *cytomapper* (v1.16.0). Demographic tables were built in the R package *gtsummary* (v2.0.4).

## Results

**Figure 1:**
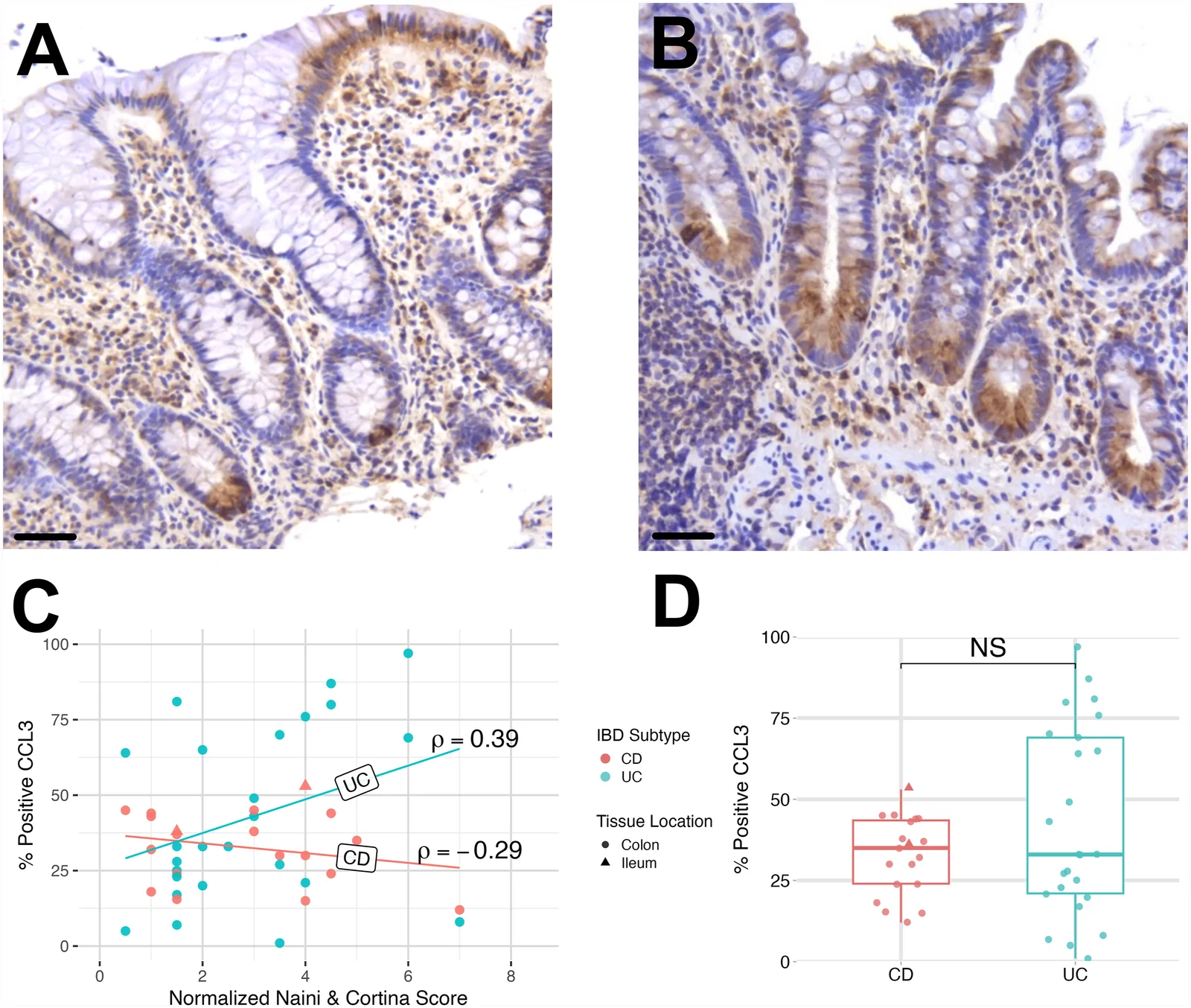
Characterization of CCL3 expression in mucosal biopsies from patients with IBD. Immunohistochemistry of CCL3 expression shows extensive but highly specific immunostaining (brown) in patients with both (A) ulcerative colitis and (B) Crohn’s disease (scale bars represent approximately 100 μm). (C) There was no significant correlation between the Naini and Cortina inflammation severity score and percent of positive CCL3 cells of UC patient GI mucosa biopsies (blue dots, Spearman’s correlation coefficient ρ = 0.39, N = 9, n = 24) or CD patient GI biopsies (red dots, Spearman’s correlation coefficient ρ = −0.287, N = 7, n = 21). (D) Wilcoxon signed-rank test indicated that there was no significant difference between the percent of positive CCL3 cells between CD and UC patients (*P* = .12, N = 15, n = 45).

To explore the expression pattern of CCL3 in IBD, IHC was performed on FFPE gut mucosa biopsies from patients, both with and without active IBD. The variety of cell types, as determined by morphologic analysis, that were positive for CCL3 were similar in both affected and unaffected patients, including neutrophils, eosinophils, lymphocytes, Paneth cells, and epithelial cells. Crypt abscess neutrophils had qualitatively more concentrated DAB staining. Epithelial cells were variably positive for CCL3, although it was predominantly cells at the base of the crypts that were positive. These patterns were similar between patients with UC (Figure 1A) and CD (Figure 1B).

The percent of CCL3-positive cells was compared to the severity of inflammation for each biopsy, as graded using the Naini and Cortina score (Figure 1C). There was no significant correlation between the percent of CCL3-positive cells and the Naini and Cortina score of UC gut mucosa biopsies (ρ = 0.39, N = 9, n = 24) or of CD gut mucosa biopsies (ρ = −0.29, N = 7, n = 21). There was no significant difference between the percent of CCL3 positive cells in CD and UC biopsies by Wilcoxon signed-rank test (Figure 1D, *P* = .12, N = 15, n = 45). Furthermore, there was no significant difference in the percent of CCL3-positive cells based on pathologist evaluation of acute disease activity, chronic changes, or the presence of any pathologic abnormality (ie, either acute or chronic inflammatory changes or both, Supplementary Figure 1A–C).

**Figure 2:**
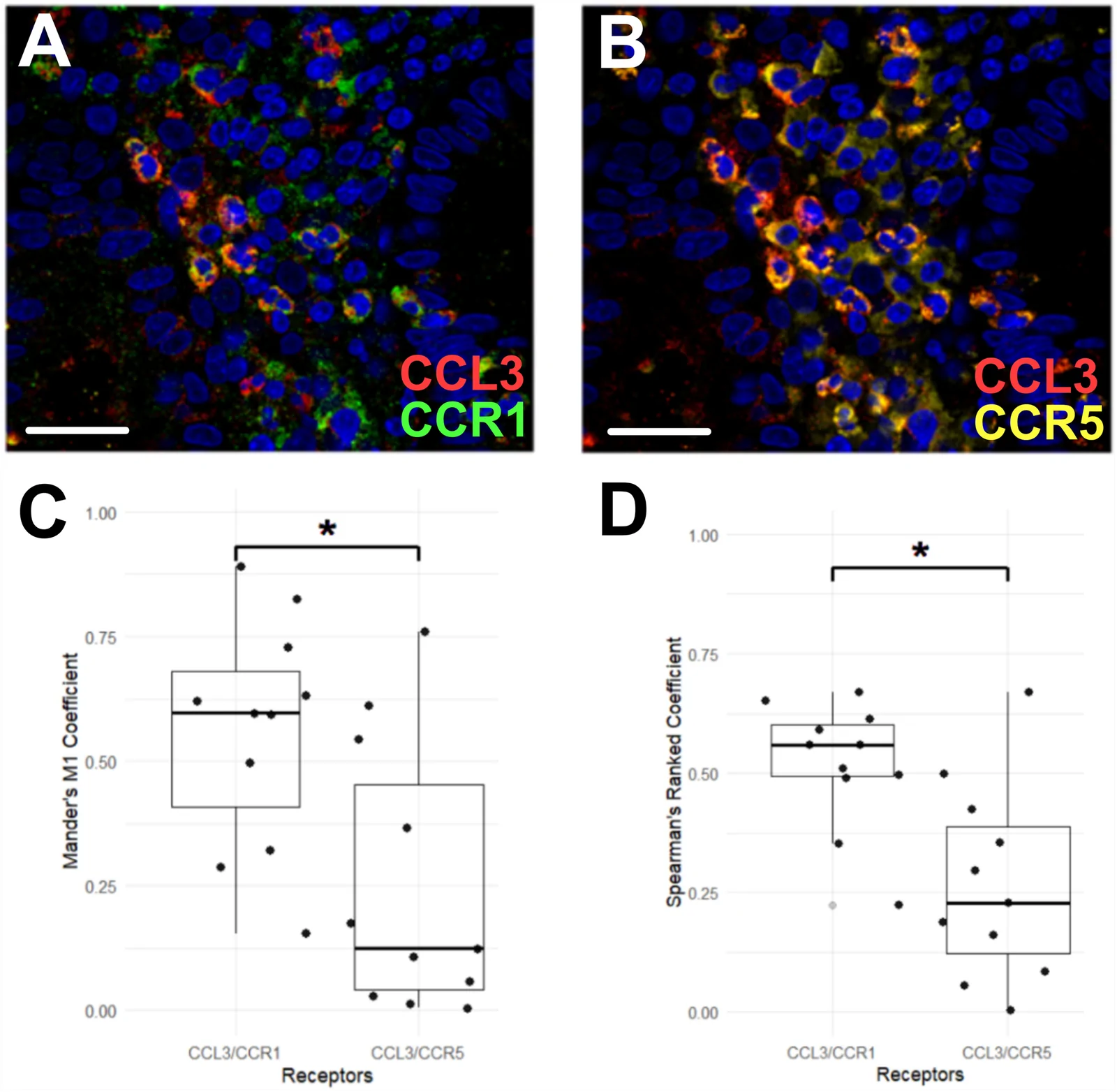
CCL3 and CCR1 have increased co-expression compared to CCL3 and CCR5 in IBD. (A) Representative image of dual immunofluorescent staining for CCL3 (red) and CCR1 (green) in an active IBD patient gut mucosa biopsy (nuclear staining with DAPI in blue). (B) Representative image of dual immunofluorescent staining for CCL3 (red) and CCR5 (yellow) in an active IBD patient gut mucosa biopsy (nuclear staining with DAPI in blue). (C) Semi-quantitative analysis of co-expression Wilcoxon signed-rank test indicated that Mander’s M1 coefficient of CCL3 with CCR1 was significantly higher than that of CCL3 with CCR5, *P* = .0128 (∗). (D) Additionally, the mean Spearman’s rank coefficient between CCL3 and CCR1 was also significantly increased than that of CCL3 with CCR5, *P* = .00947 (∗). DAPI, 4′,6-diamidino-2-phenylindole.

Immunofluorescent (IF) costaining for CCL3 and its putative receptors, CCR1 and CCR5, was performed on pediatric FFPE gut mucosa biopsies of patients with both active IBD and those in remission (Figure 2A and B). Both co-occurrence and correlation of CCL3 with its receptors were measured using Mander’s M1 coefficient and Spearman’s ranked coefficient, respectively. The mean of the coefficients of individual biopsies was calculated. The proportion of CCL3 that co-occurred with CCR1 was significantly higher than the proportion of CCL3 that co-occurred with CCR5, as indicated by Mander’s M1 coefficient (Figure 2C). Furthermore, CCL3 intensity correlated significantly more with the intensity of CCR1 than it did with CCR5, as indicated by Spearman’s ranked coefficient for biopsy (Figure 2D). The mean fluorescent intensities of CCL3, CCR1, and CCR5 were compared between the degree of histologic activity, chronicity, presence of any pathologic abnormality, and IBD subtype. There were no significant differences between the mean fluorescent intensities of CCR1 or CCR5 based on pathology activity, chronicity, or presence of any pathologic abnormality. Nor was there a difference between expression of these markers between UC and CD biopsies. Furthermore, there was no correlation between the severity of inflammation of each biopsy, as graded with the Naini and Cortina score, and the mean fluorescent intensities of CCR1 or CCR5.

The co-occurrence and correlation of CCL3 with CCR1 and CCR5 were compared based on the severity of disease activity, chronicity, presence of any abnormality, and subtype. There were no significant differences between the co-occurrence of CCL3 with CCR1 or the co-occurrence of CCL3 with CCR5-based pathology activity, chronicity, the presence of a pathology abnormality, or between UC and CD gut biopsies. There was no significant difference between the correlation of intensities of CCL3-CCR1 and CCL3-CCR1-based pathologic activity, chronicity, the presence of a pathologic abnormality, or between UC and CD gut biopsies.

**Figure 3:**
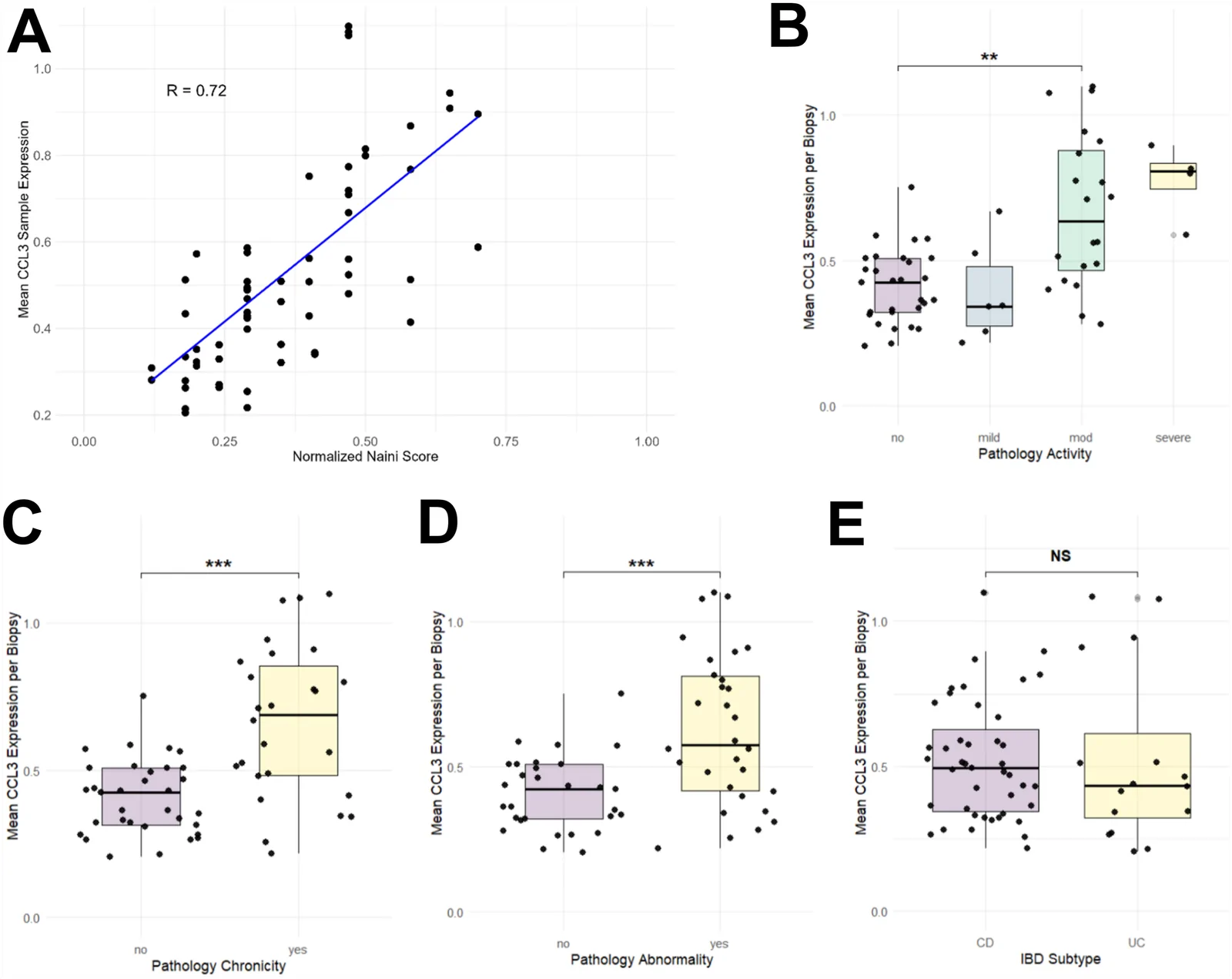
Imaging mass cytometry (IMC) shows CCL3 expression correlates with degree of inflammation. (A) Mean (per sample) CCL3 expression (relative mean intensity) from IMC analysis of each sample was positively correlated with the normalized Naini and Cortina score of inflammation (Spearman’s correlation coefficient ρ = 0.72, n = 59). (B) There was a significant difference in CCL3 expression across the severity categories of active disease of biopsies (Kruskal-Wallis test, X2 = 12.913, *P* = .000247, n = 59). Specifically, Dunn’s multiple comparisons test indicated that there was a significant difference between “moderate” (n = 20 biopsies) and “no” (n = 29 biopsies) pathology activity, *P* = .0035, ∗∗. (C) CCL3 expression in IBD patient biopsies with chronic inflammatory changes (n = 30 biopsies) was higher than in biopsies with no chronic changes (n = 26) (Wilcoxon signed-rank test, *P* = 2.2∗10–16, ∗∗∗). (D) Mean CCL3 expression in biopsies with any IBD-associated pathology abnormality (acute or chronic inflammatory changes) (n = 33 biopsies) was higher than biopsies with no pathology chronicity (n = 26) (Wilcoxon signed-rank test, *P* = 2.2∗10–16, ∗∗∗). (E) There was no significant difference between mean CCL3 expression in biopsies of UC (n = 16) and CD (n = 44) patients (Wilcoxon signed-rank test, *P* = .62, NS).

Following characterization and evaluation of CCL3 expression by IHC, we investigated CCL3 spatial relationships at the single cell via IMC, which allowed for specific identification of CCL3-expressing cell types and their spatial relationship to other relevant immune cell types. As seen in Figure 3A, the Spearman’s ranked correlation, ρ = 0.72, indicated a positive correlation between the Naini and Cortina score and CCL3 expression levels in IBD biopsies (both active and remission). The correlation was driven by increased CCL3 expression in patients with moderate and severe inflammatory changes, and relatively lower CCL3 expression was seen in patients with mild or no inflammatory changes (Figure 3B). Mean CCL3 expression was significantly higher in biopsies with moderate acute inflammation compared to those with no acute inflammation, *P* = .0035. Mean CCL3 expression was higher in biopsies with chronic inflammation compared to no chronic inflammation, *P* = 2.2∗10^−16^ (Figure 3C). CCL3 expression was also significantly higher in biopsies with any (acute or chronic) pathologic abnormality, *P* = 2.2 ∗10^−16^ (Figure 3D). There was no significant difference between CCL3 expression in biopsies from patients with UC compared to CD (Figure 3E). For the patient that had biopsies from both inflamed and non-inflamed regions, the CCL3 levels in both biopsies were comparable and within the central region of the overall distribution and were not outliers in their respective groups.

**Figure 4:**
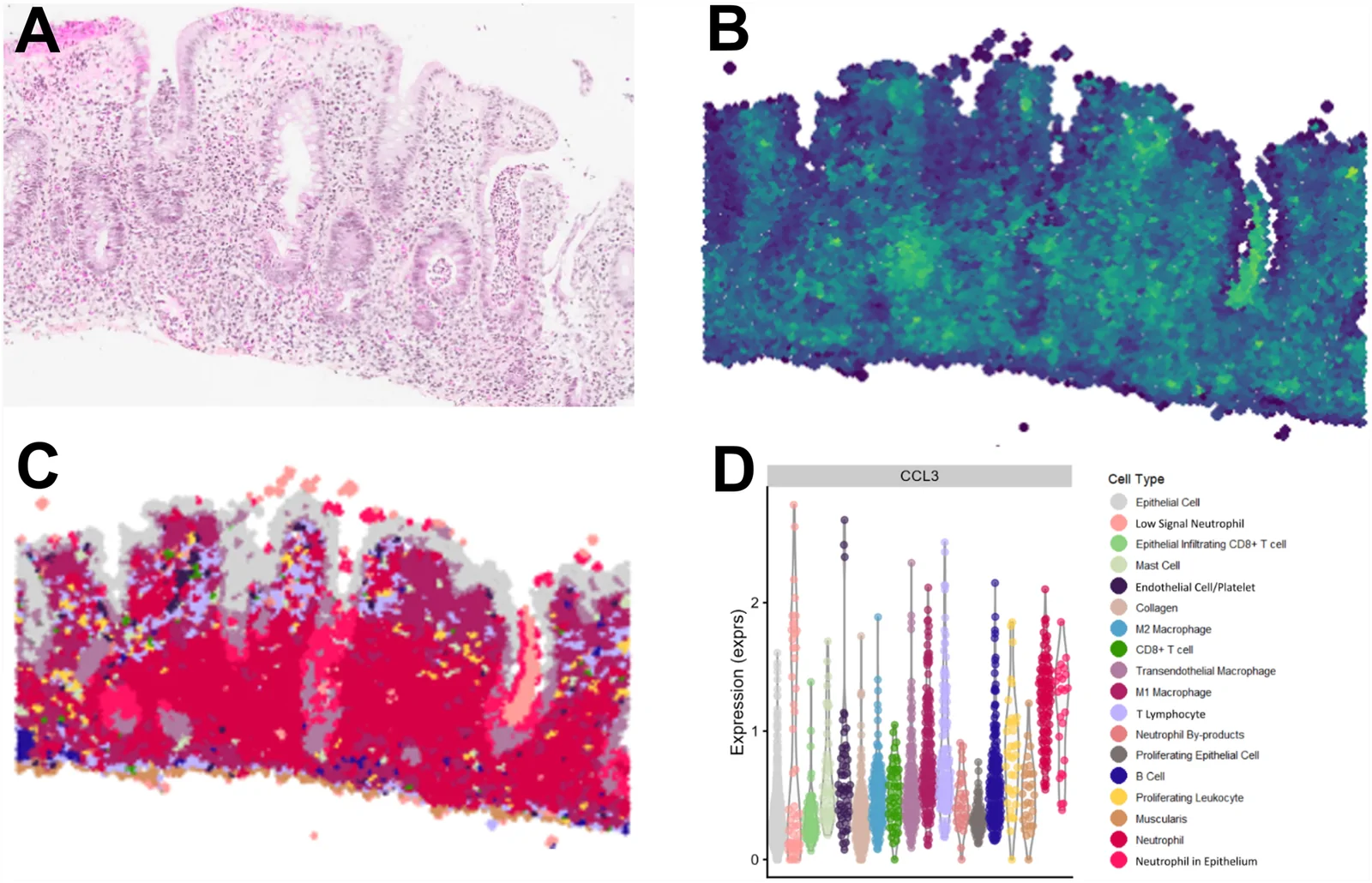
CCL3 expression is localized in neutrophil and M1 macrophage clusters in the gut mucosa in patients with IBD. (A) Representative image of a severely inflamed gut mucosal biopsy from a patient with Crohn’s disease (hematoxylin and eosin). (B) Scaled CCL3 expression by cell by imaging mass cytometry (IMC) of the same region of interest as in panel A, showing localization of CCL3 expression (high expression is green, low expression is blue), especially in the lamina propria and in a neutrophil crypt abscess (right-most crypt in image). (C) IMC recreation image with each cell colored by cell type assignment (legend in panel D) for each cell in the region of interest corresponding to the same location in panels A and B. (D) Global CCL3 expression by cell type across all IMC samples (n = 29), demonstrating higher expression especially in neutrophil and M1 macrophage clusters.

To further investigate CCL3 spatial relationships in IBD patient biopsies, images were compared of routine (hematoxylin and eosin) stained images (Figure 4A), CCL3 expression by IMC (Figure 4B), and cell-type identification by IMC (Figure 4C). CCL3 expression was high in regions of clustered neutrophils. CCL3 was consistently expressed in all regions of neutrophil localization but was highest in regions where neutrophils were found in the crypt lumen. CCL3 expression was also high in regions where M1 macrophages clustered. In contrast to neutrophil CCL3 expression, in regions of M1 macrophage localization CCL3 was inconsistently expressed. CCL3 expression was also high in regions of naive B cell localization. CCL3 was also variably expressed in epithelial cells, similar to the pattern seen via IHC. Levels of CCL3 expression across all cell types are shown in Figure 4D. There were many cell types beyond the classically described neutrophils and macrophages that expressed CCL3 in the gut mucosa during IBD. Across the 3 cohorts investigated throughout this study, CCL3 levels from paired biopsies from the same patients did not consistently cluster together.

## Discussion

This study aimed to characterize the presence of the chemokine CCL3 in the GI mucosa in patients with IBD inflammation. CCL3 expression in IBD patient gut mucosa samples was compared across disease activity states, IBD subtypes, and specific cell types. While previous studies have shown CCL3 expression in the GI mucosa, this is the first study to characterize its role in IBD at the single-cell level. CCL3 was found to be expressed in a variety of cell types including those previously described, such as neutrophils and M1 macrophages, as well as in novel cell types in this tissue, including epithelial cells and naive B cells.

IHC was used to identify a variety of cell types in the GI mucosa expressing CCL3. Proteomic analysis found that there was a positive correlation between CCL3 expression levels and the severity of inflammation. These findings are similar to those of Lai et al,^8^ who found that CCL3 expression is higher in gut biopsies from patients with UC compared to healthy patients. Furthermore, Lopetuso et al^6^ found that CCL3 is more highly expressed in inflamed than in non-inflamed biopsies from UC patients. In the present study, there was no significant difference between CCL3 expression between UC and CD patient biopsies. This differs from the observations made by Banks et al^10^, who found that CD biopsies had higher CCL3 expression than UC biopsies. Notably, the present study includes more IBD patients and utilizes multiplex IMC, which offers stoichiometric linearity and a wider dynamic range compared to conventional IHC. The current study also incorporates an objective score of inflammation for improved understanding of the degree of inflammation in each biopsy.

Similarly to previous studies published by Banks et al^10^ and Grimm et al,^11^ this study showed that CCL3 expression was highly expressed by inflammatory cells. Neutrophils and M1 macrophages were among the highest-expressing cells for CCL3. There was also positive staining in the epithelial barrier, which has not been previously well characterized, although this finding was also observed by Banks et al.^10^ The role of CCL3 expression in the epithelium is not yet characterized and would be an important subject for future studies. Paneth cells also appeared to be positively stained for CCL3 expression, although the identification of Paneth cells was possible only by morphology in this study. This cell type has not been previously described as expressing CCL3, presumably because of preferential use of colon biopsies in previous studies. In healthy conditions, Paneth cells are found in the small intestine and can be found at the base of crypts. Their role is to produce antimicrobial peptides in the crypts.^17^ Though characterized by their antimicrobial defense, there is evidence of other chemokine and pro-inflammatory cytokine production by Paneth cells.^18^ Given that Paneth cells and epithelial cells are always present in the gut mucosa, if further studies can confirm their production of CCL3, these cells may be a part of an early phase of inflammation in IBD, prior to the majority of inflammatory cell arrival. Future studies with positive and negative staining controls will be required to further understand this potential mechanism.

Dual immunofluorescent staining showed that there was expression in the GI mucosa of both putative receptors for CCL3: CCR1 and CCR5. Dual expression of the chemokine and its receptor was found in some cells, while others expressed only one or other. The co-occurrence of CCR1 with CCL3 was higher than that of CCR5 to CCL3 expression. These findings point to a potential complex regulatory mechanism for CCL3 activity that could be driven by specific cell types with variable expression of this chemokine system. Lai et al^8^ found that the CCL3-CCR1 communication pathway has more potential for macrophage signaling than the CCL3-CCR5 pathway. It is possible that CCL3 plays an important role in the initiation of the inflammatory response, drawing early inflammatory cell types to the site of inflammation, such as neutrophils and M1 macrophages. These cells could then produce further CCL3, potentially playing a role in the ongoing inflammatory activity of IBD. This could differ from other inflammatory states such as infection that appear to be self-limited by immune regulatory processes, which are often dysfunctional in IBD. This finding is similar to Dobre et al^19^ and Ye et al^20^ who found that CCR1 and CCR5 expression was increased in active relative to remission biopsies, respectively. Using IMC, CCL3 expression was found to be highest in neutrophils and pro-inflammatory macrophages. These findings align with previous findings that CCL3 is primarily associated with macrophages and neutrophils.^4^^,^^21^, ^22^, ^23^

This study, as with all clinical cohort studies, is limited by the heterogeneity of IBD patients. Because of the nature of IBD, most patients are undergoing treatment when the biopsies are taken, and as a result, it is difficult to eliminate treatment as a confounding variable when analyzing CCL3 expression. This was partially addressed in our study but including mostly patients that were newly diagnosed at the time of their biopsies and thus were treatment naïve. Non-IBD patients were not included in our study, as previous studies have already determined that CCL3 is more highly expressed in IBD than healthy biopsies, which is why this study focused on differences between inflammatory activity states between IBD patients instead.^10^^,^^11^

In this study, dual IF was performed to determine the relationship between CCL3 and its receptors, CCR1 and CCR5. However, this technique is limited by the fact that it cannot be determined if areas of dual staining represent cells co-expressing these proteins or receptor-ligand binding or both. To accurately identify whether the CCL3 ligand is binding to its receptors, one potential approach could be to measure receptor downstream signaling proteins in the presence of CCL3 as a functional output of potential ligand interaction. Additionally, receptor-ligand assays can be developed, although such an experiment is beyond the scope of this project.

It is not possible from the current study results to determine whether a cell expresses CCL3 or has CCL3 bound to a cell membrane receptor with the techniques used in this study. Spatial transcriptomics or in situ hybridization would be needed to confirm that a cell is transcribing CCL3 rather than binding exogenously produced CCL3. This would also confirm which cell lineages are more likely to express CCL3 and which are polarized by CCL3 in the context of IBD. Future studies using spatial transcriptomics or in situ hybridization could also confirm whether CCL3-receptor interaction is increased in active cases of IBD. It will also be important to identify the specific cell types in this pathway in future studies. This could identify anti-CCL3 or anti-CCL3-receptor-targeted therapeutic interventions for potentially treating IBD. Additionally, a study evaluating intestinal inflammation after administering CCL3 in an experimental model of IBD or those with a genetic deficiency for CCR1 or CCR5 coupled with IBD models could support whether an anti-CCR1 or anti-CCR5 therapeutic could be more effective in decreasing inflammation in IBD. Additionally, although CCL3 expression may vary by anatomical location between the colon and terminal ileum, the limited and uneven representation of ileal and colonic biopsies in this cohort and sample size precluded formal comparison between sites.

## Conclusion

It appears that CCL3 plays an important role in driving the inflammatory response in IBD and thus may be a potential therapeutic target for active IBD in specific subsets of patients. Spatial proteomics suggests that both neutrophils and M1 macrophages are crucial to driving the CCL3 component of IBD activity and that the CCL3-CCR1 signaling pathway has more potential communication in the context of IBD than the CCL3-CCR5 signaling pathway. Confirmation of these findings with the use of spatial transcriptomics will support the investigation of CCL3 as a potential therapeutic target to treat IBD-induced inflammation.

## Supplementary Materials

Supplementary Materials

Extended PDF
