# Supplementary material for: Chemokine Ligand-3 (CCL3) as a Novel Mediator of Inflammatory Bowel Disease Activity
Source: Gastro Hep Adv. 2026 Aug 4;5(11):101081. doi: 10.1016/j.gastha.2026.101081 (PMC13546898; doi:10.1016/j.gastha.2026.101081)
Supplement: Supplementary file 1 [file mmc1.pdf]

Supplementary Materials for O'Brien et al. "Chemokine Ligand-3 (CCL3) as a Novel Mediator of Inflammatory Bowel Disease Activity"

Table of Contents

Supplementary Table 1: Patient characteristics.

Supplementary Table 2: Antibodies used for immunohistochemistry, immunofluorescence, and imaging mass cytometry experiments.

Supplementary Table 3: Parameter settings for positive cell detection in QuPath.

Supplementary Table 4. Antibodies used for imaging mass cytometry.

Supplementary Table 5: Cluster identities and expression of cell markers.

Supplementary Figure 1: Naini & Cortina score assigned to biopsies correlate with pathologist's assessment.

Supplementary Figure 2: UMAP dimensional reduction of IMC marker expression based on patient ID and IBD subtype.

Supplementary Figure 3: Validation of cluster parameter combinations.

Supplementary Figure 4: Histogram of CCL3+ cells expression per cell.

Supplementary Table 1. Patient characteristics for biopsy samples (N values refers to number of biopsies) used in: (A) immunohistochemistry experiments, (B) immunofluorescence experiments, and (C) imaging mass cytometry experiments. ADL, adalimumab; ASA, 5-aminosalycilic acid; AZA, azathioprine; BUD, budesonide; CD, Crohn's disease; MTX, methotrexate; PRED, prednisone; UC, ulcerative colitis VEDO, vedolizumab.

Table A. IHC Data Patient Characteristics

	Pathology Abnormality	
**Variable**	**n**<br>N = 15[1]	**y**<br>N = 30[1]
**Age at Scope**	38 (11)	38 (15)
**Sex**		
female	11 / 15 (73%)	27 / 30 (90%)
male	4 / 15 (27%)	3 / 30 (10%)
**Medication**		
ADL	3 / 15 (20%)	1 / 30 (3.3%)
ASA	3 / 15 (20%)	11 / 30 (37%)
AZA	1 / 15 (6.7%)	3 / 30 (10%)
BUD	0 / 15 (0%)	1 / 30 (3.3%)
MTX	1 / 15 (6.7%)	1 / 30 (3.3%)
NONE	5 / 15 (33%)	10 / 30 (33%)
PRED	0 / 15 (0%)	2 / 30 (6.7%)
VEDO	2 / 15 (13%)	1 / 30 (3.3%)
**Subtype**		
CD	10 / 15 (67%)	11 / 30 (37%)
UC	5 / 15 (33%)	19 / 30 (63%)
**Naini Score**	3 (1)	6 (3)

[1] Mean (SD); n / N (%)

Table B. IF Data Patient Characteristics

	Pathology Abnormality	
**Variable**	**n**<br>N = 5[1]	**y**<br>N = 6[1]
**Age at Scope**	14 (3)	13 (3)
**Sex**		
female	1 / 5 (20%)	4 / 6 (67%)
male	4 / 5 (80%)	2 / 6 (33%)
**Medication**		
NONE	5 / 5 (100%)	6 / 6 (100%)
**Subtype**		
CD	4 / 5 (80%)	3 / 6 (50%)
UC	1 / 5 (20%)	3 / 6 (50%)
**Naini Score**	2 (2)	9 (1)

[1] Mean (SD); n / N (%)

Table C. IMC Data Patient Characteristics

	Pathology Abnormality	
**Variable**	**n**<br>N = 15[1]	**y**<br>N = 15[1]
**Age at Scope**	16 (10)	16 (9)
**Sex**		
female	7 / 15 (47%)	7 / 15 (47%)
male	8 / 15 (53%)	8 / 15 (53%)
**Medication**		
ASA	1 / 15 (6.7%)	1 / 15 (6.7%)
AZA	1 / 15 (6.7%)	0 / 15 (0%)
NONE	13 / 15 (87%)	14 / 15 (93%)
**Subtype**		
CD	11 / 15 (73%)	11 / 15 (73%)
UC	4 / 15 (27%)	4 / 15 (27%)
**Naini Score**	4 (1)	7 (2)

[1] Mean (SD); n / N (%)

Supplementary Table 2. Antibodies used for immunohistochemistry and immunofluorescence experiments.

**Antibody**	**Company/ID#**	**Specificity/Clone**
Anti-human CCL3	Abcam 32609	Rabbit Polyclonal IgG
Anti-human CCR5	Novus 45523	Mouse Monoclonal IgG2b
Anti-human CCR1	Abcam 139399	Goat Polyclonal IgG
Donkey Anti-Goat IgG H&L Alexa Fluor 488 (green)	Abcam 150129	Goat IgG H+L
Donkey Anti-Rabbit IgG H&L Alexa Fluor 647 (red)	Abcam 15007	Rabbit IgG H+L
Donkey Anti-Mouse IgG H&L Alexa Fluor 555 (yellow)	Abcam 150106	Mouse IgG H+L
Anti-human CD3	StandardBio PN# 3170019D	Polyclonal, C-terminal
Anti-human CD45RO	StandardBio PN#3173016D	UCHL1
Anti-Human CD68	StandardBio PN#3159035D	KP1
Anti-E-Cadherin	StandardBio PN#3158029D	24E10

Anti-Human CD4	StandardBio PN#3156033D	EPR6855
Anti-Human CD8a	StandardBio PN#3162034D	C8/144B
Anti-Human Pan Keratin	StandardBio PN#3148020D	C11
Human S100A9 antibody	R&D Systems Cat# AF5578	Sheep Polyclonal IgG
CCL3 antibody	ThermoFisher Cat# PA5-47000	Goat Polyclonal IgG
Human/Mouse MPO antibody	R&D Systems Cat# AF3667	Goat Polyclonal IgG
CD20 antibody	StandardBio PN#201506	H1
FoxP3	StandardBio PN#201506	PCH101

Supplementary Table 3. Parameter settings for DAB positive cell detection in QuPath (version 0.4.4) for the detection of DAB/CCL3 positive cells in IBD patient gut biopsies.

**Detection image**	Hematoxylin OD
**Requested Pixel size**	254.5833 µm
**Background Radius**	1000 µm
**Median filter radius**	0 µm
**Sigma**	200 µm
**Minimum area**	1000000 $µm^2$
**Maximum area**	7000000 $µm^2$
**Threshold**	0.06
**Max Background Intensity**	2
**Cell Expansion**	800 µm
**Score compartment**	Cell: DAB OD mean

Supplementary Table 4. Antibodies used for imaging mass cytometry.

Metal	Marker	Clone	Supplier
**89Y**	Vimentin	D21H3	SBT
**141Pr**	aSMA	1A4	SBT
**142Nd**	CD16	SP175	Abcam
**143Nd**	Ki-67	B56	BD
**144Nd**	CD14	EPR3653	SBT
**145Nd**	MPO	EPR20257	Abcam
**146Nd**	CD206	E2L9N	CST
**147Sm**	CD163	EDHu-1	SBT
**148Nd**	Pan-cytokeratin	C11	SBT
**149Sm**	CD15	W6D3	SBT
**150Nd**	MUC1	SM3	SBT
**151Eu**	CCL3	Polyclonal	ThermoFisher
**152Sm**	CD45	D9M8I	SBT
**153Eu**	CD117	YR145	Abcam
**154Sm**	CD11c	EP1347Y	SBT
**155Gd**	Foxp3	236A/E7	SBT
**156Gd**	CD4	EPR6855	SBT
**158Gd**	E-cadherin	24E10	SBT
**159Tb**	CD68	KP1	SBT
**160Gd**	MMP9	EP1255Y	Abcam
**161Dy**	CD20	H1	SBT

**162Dy**	CD8a	C8/144B	SBT
**163Dy**	CD11b	EPR1344	SBT
**165Ho**	β-catenin	D13A1	SBT
**166Er**	CD45RA	HI100	SBT
**167Er**	CD169	SP216	Abcam
**168Er**	CD40	EPR20540	Abcam
**169Tm**	Collagen I	Polyclonal	SBT
**170Er**	CD3	Polyclonal	SBT
**171Yb**	S100A9	Polyclonal	R&D Systems
**173Yb**	CD45RO	UCHL1	SBT
**174Yb**	HLA-DR	EPR3692	Abcam
**176Yb**	CD31	EP3095	Abcam
**191Ir**	DNA1	N/A	SBT
**193Ir**	DNA2	N/A	SBT

SBT, Standard Biotools

Cluster	Given Identify	Marker expression
1	Epithelial Cell	E-Cad^{pos}B-Catenin^{pos}Pan-CK^{pos}
2	Low Signal Neutrophil	E-Cad^{neg}B-Catenin^{neg}Pan-CK^{neg}CD15^{pos}CD3^{neg}CD68^{neg}S100A9^{low}MPO^{low}CCL3^{low}
3	Epithelial Cell	E-Cad^{pos}B-Catenin^{pos}Pan-CK^{pos}
4	Epithelial Infiltrating CD8+ T cell	E-Cad^{pos}B-Catenin^{pos}Pan-CK^{pos}CD8a^{high}CD3^{pos}CD45^{pos}
5	Epithelial Cell (muscle boarder)	E-Cad^{pos}B-Catenin^{pos}Pan-CK^{pos}CollagenI^{high}aSMA^{high}
6	Mast Cell	CD117^{high}CD68^{mod}CD3^{low}CD15^{neg}
7	Endothelial Cell /Blood vessel	CD31^{high}CCL3^{low}CollagenI^{low}Vimentin^{mod}CD3^{neg}CD68^{neg} CD15^{neg}E-Cad^{neg}Pan-CK^{neg}B-Catenin^{neg}
8	Collagen	CollagenI^{mod}CD3^{neg}CD68^{neg}CD15^{neg}E-Cad^{neg}Pan-CK^{neg}B-Catenin^{neg}
9	M2 Macrophage	CD68^{high}CD163$_{high}$CD169^{mod}CD206^{high}HLA-DR^{low}CD4^{mod}
10	CD8+ T Cell	CD8a^{high}CD45RO^{high}CD3^{mod}CD45^{mod}CD45RO^{low}
11	Transendothelial Macrophage	E-Cad^{pos} B-Catenin^{pos}Pan-CK^{pos} Ki67^{pos}MUC1^{pos}CD16^{pos}CD14^{pos}CD68^{pos}

12	M1 Macrophage	$CD68^{high}CD45RO^{high}CD45^{high}S100A9^{high}CD163^{mod}CD169^{mod}CD206^{mod}$HLA-$DR^{mod}MMP9^{mod}CD40^{mod}MPO^{low}CD3^{low}CD4^{low}CD11b^{low}CD11c^{low}CD16^{high}CD15^{neg}$
13	T lymphocyte	$CD3^{low}$ $CD4^{low}CD68^{neg}CD15^{neg}$E-$Cad^{neg}$Pan-$CK^{neg}$B-$Catenin^{neg}CD31^{mod}CD45RA^{mod}CCL3^{mod}$
14	Neutrophil by-product (MPO integrin)	$CD3^{neg}CD68^{neg}CD15^{neg}$E-$Cad^{neg}$Pan-$CK^{neg}$B-$Catenin^{neg}MPO^{mod}CD11b^{mod}$ $S100A9^{low}CD45^{pos}$
15	Proliferating Epithelial Cell	E-Cad^{pos} B-$Catenin^{pos}$Pan-$CK^{pos}MUC1^{pos}Ki67^{high}CD3^{neg}CD68^{neg}CD15^{neg}$
16	Epithelial Cell	E-Cad^{pos} B-$Catenin^{pos}$Pan-$CK^{pos}MUC1^{pos}CD3^{neg}CD68^{neg}CD15^{neg}CD14^{low}$
17	B Cell	$CD20^{high}CD45RA^{high}CD3^{neg}FoxP3^{neg}CD11c^{neg}$
18	Proliferating Leukocyte	$Ki67^{high}CD3^{pos}$ $CD45^{pos}$ $CD15^{neg}$ $CD20^{neg}$ E-Cad^{neg}, Pan-CK^{neg}, B-$Catenin^{neg}$, all else is at low expression
19	Muscularis	$aSMA^{high}$ $CD16^{mod}$
20	Neutrophil	$CD15^{high}MMP9^{high}MPO^{high}S100A9^{high}CD11b^{high}CCL3^{high}$
21	Neutrophil in Epithelium	$CD15^{high}MMP9^{high}MPO^{high}S100A9^{high}CD11b^{high}CCL3^{high}$ E-Cad^{pos}B-$Catenin^{pos}$Pan-CK^{pos}HLA-DR^{high}

Supplementary Table 5. Cluster identities and expression of cell markers.

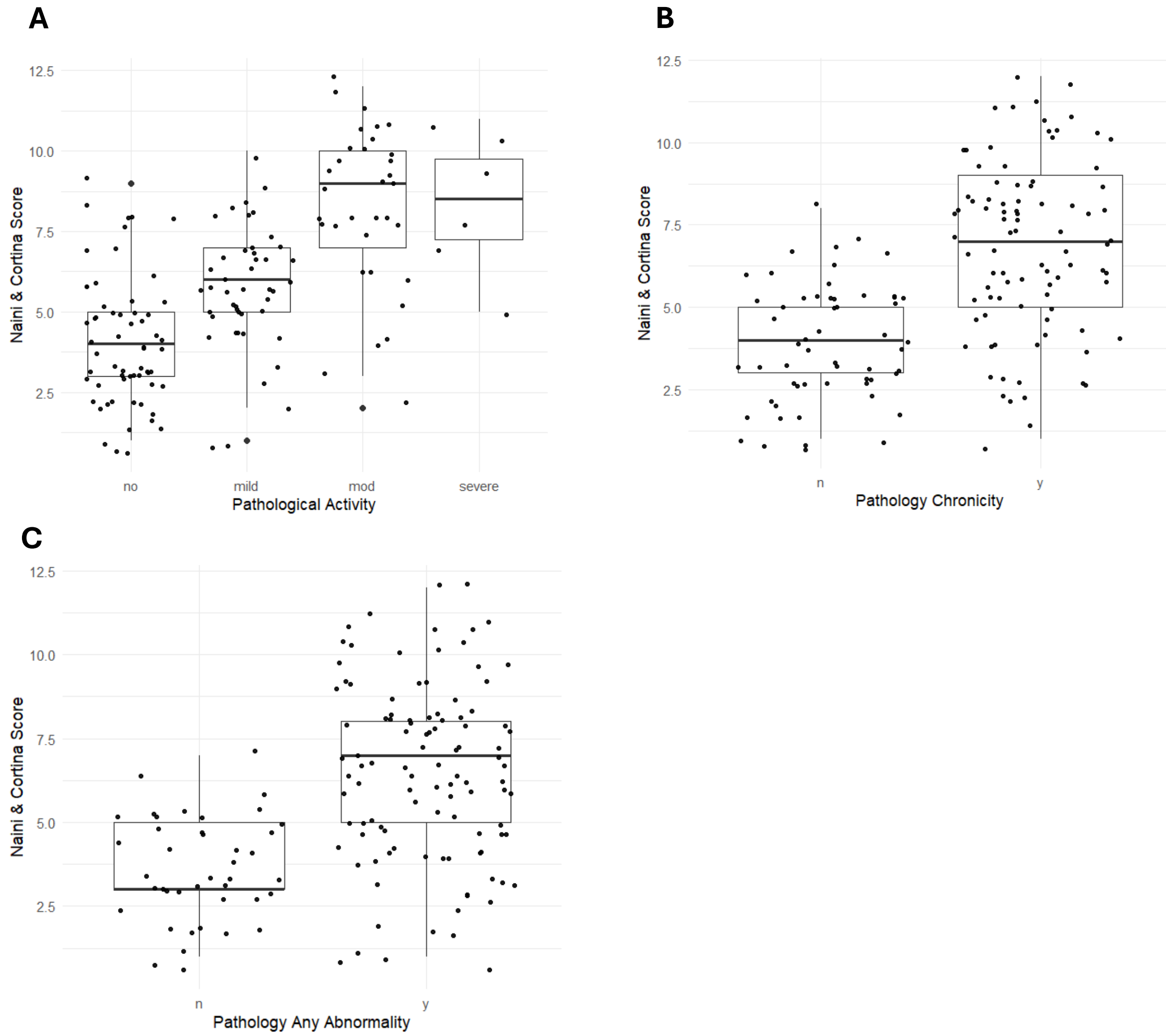

Supplementary Figure 1. Naini & Cortina score correlates with pathologist's assessment. (A) Naina & Cortina score and inflammation severity as scored by pathologist assignment. **(B)** Naini & Cortina score in biopsies with absent ("n") or present ("y") chronic inflammation. **(C)** Naini & Cortina score in biopsies with the absence ("n") or presence ("y") of any form of inflammation (either chronic or acute or both).

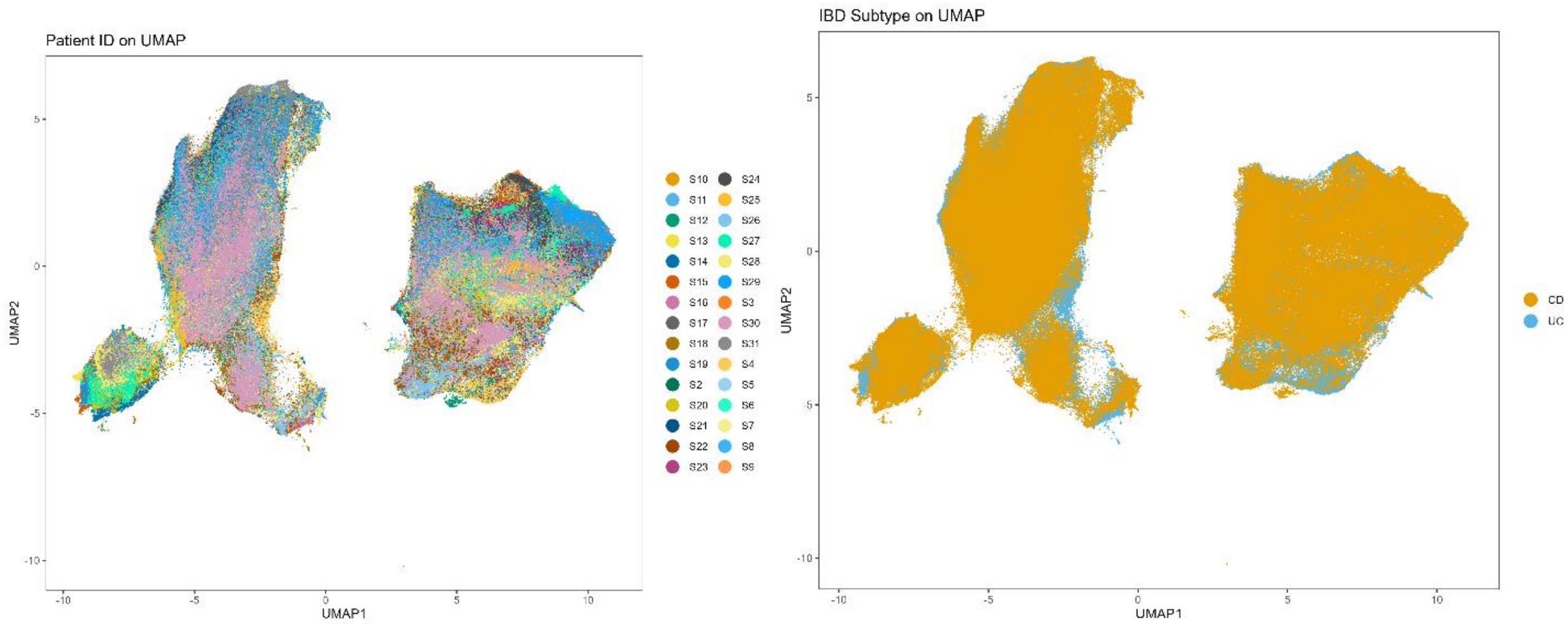

Supplementary Figure 2. UMAP dimensional reduction of IMC marker expression based on patient ID and IBD subtype. (A) Marker expression based on patient sample ID or (B) on IBD disease subtype (orange – Crohn's disease; blue – ulcerative colitis).

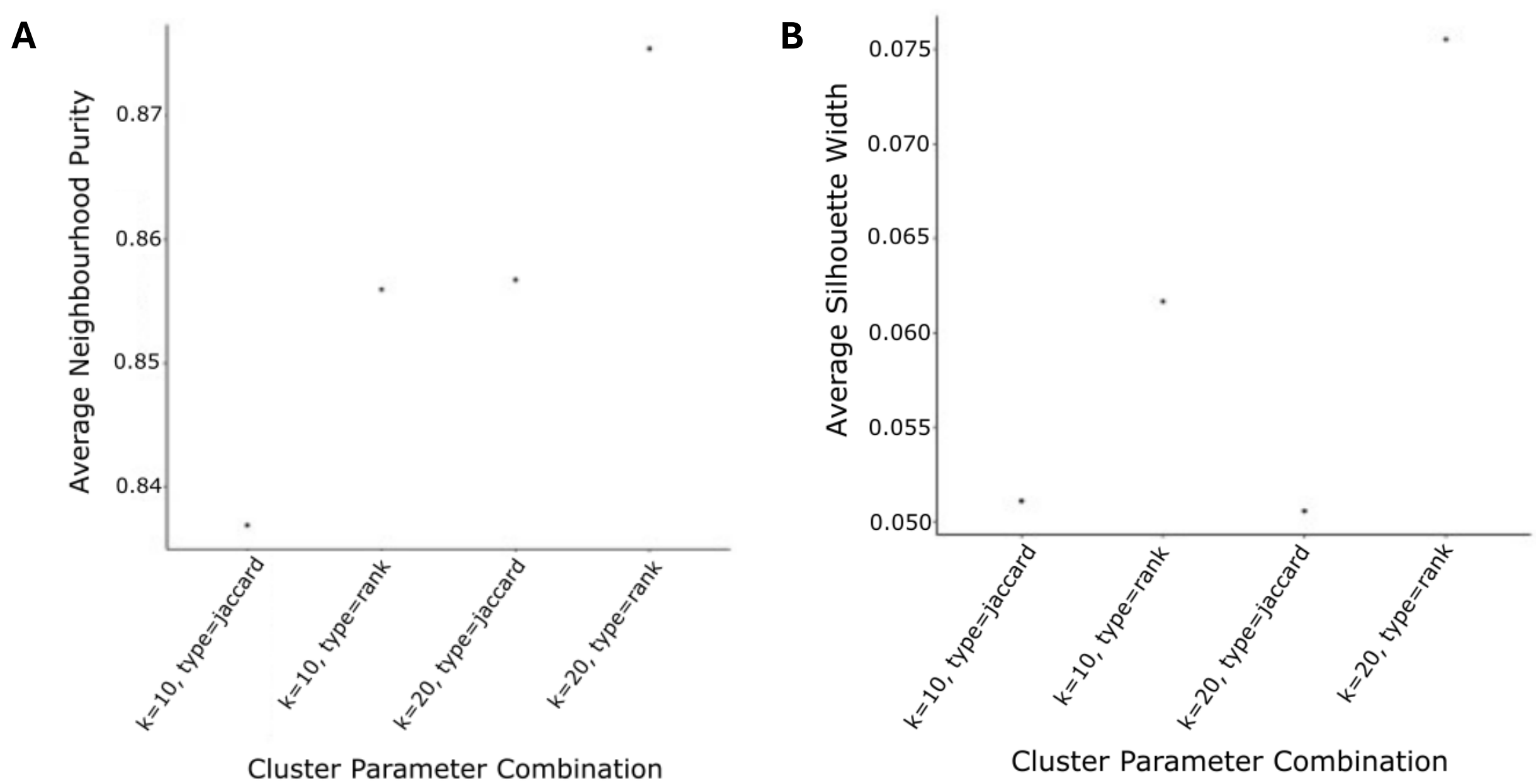

Supplementary Figure 3. Validation of cluster parameter combinations. **(A)** Average neighbourhood purity with different cluster parameter combinations. Based on this graph, k=20 and type=rank (last parameter combination on x-axis) were selected as the optimal parameters with the highest neighbourhood purity. **(B)** Average silhouette width with different cluster parameter combinations. A cell with a large positive width is closer to other cells from the same cluster compared to cells from different clusters. Based on this figure, k=20 and type=rank (last parameter combination on x-axis) were also selected as the optimal parameters for clustering.

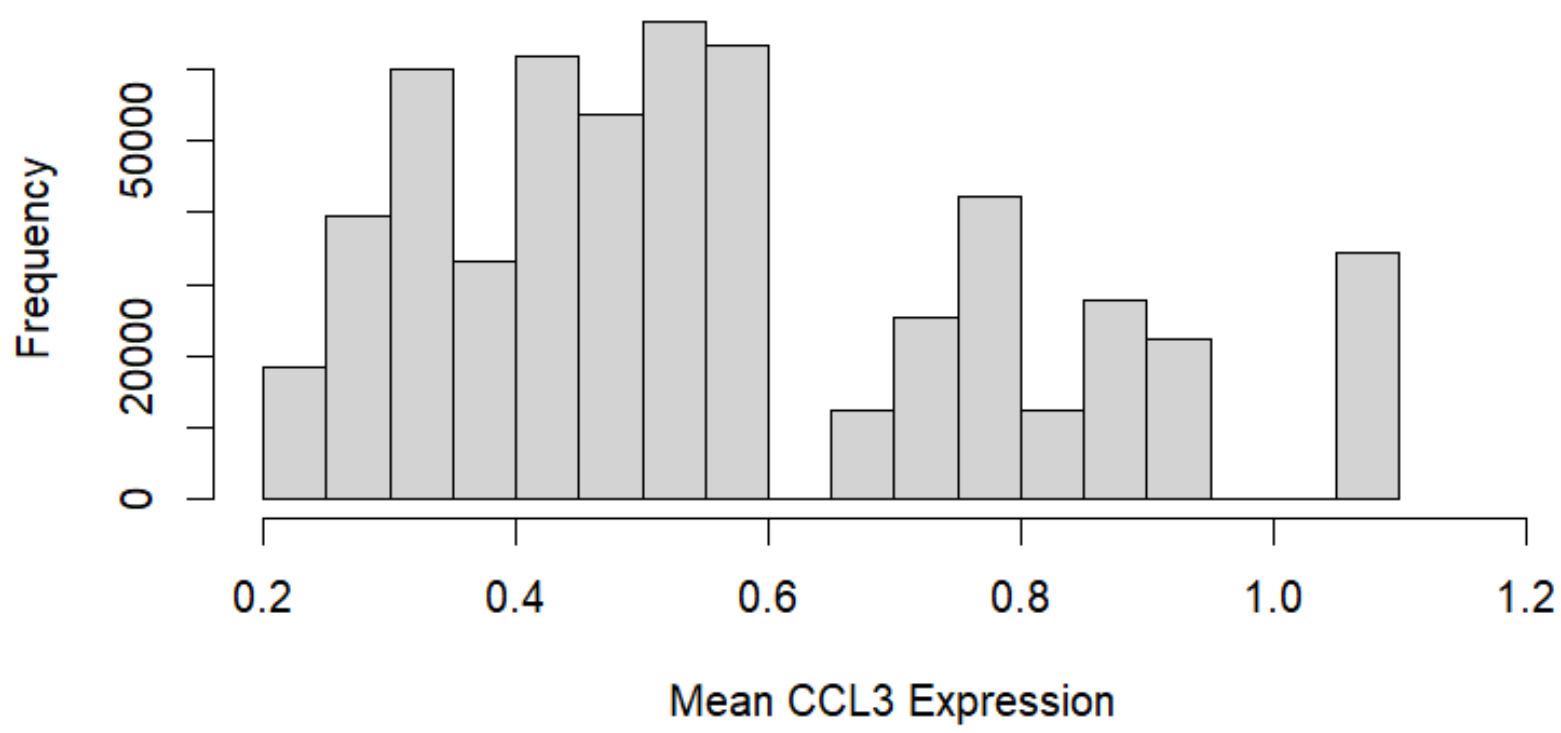

Supplementary Figure 4. Histogram of mean CCL3+ cells expression per cell. Mean CCL3 expression per cell (x-axis) was not normally distributed across all positive cells (counts on y-axis).
